# Development and quality assessment of gluten-free cookies using rice flour and date fruit

**DOI:** 10.3389/fnut.2025.1645063

**Published:** 2025-09-02

**Authors:** Sajeela Akram, Aqsa Chatha, Juweria Abid, Umar Farooq, Abdul Momin Rizwan Ahmad

**Affiliations:** ^1^Department of Human Nutrition and Dietetics, University of Chakwal, Chakwal, Pakistan; ^2^Department of Food Science and Human Nutrition, University of Illinois Urbana-Champaign, Champaign, IL, United States; ^3^Department of Nutrition and Dietetics, National University of Medical Sciences, Rawalpindi, Pakistan; ^4^Department of Health Sciences, University of York, York, United Kingdom; ^5^Department of Human Nutrition and Dietetics, NUST School of Health Sciences, National University of Sciences and Technology, Sector H-12, Islamabad, Pakistan

**Keywords:** rice flour, date palm, proximate analysis, celiac disease, gluten sensitivity, gluten-free cookies, organoleptic properties

## Abstract

**Introduction:**

Bakery products formulated with wheat flour as the major constituent are unfit for consumption for people with gluten sensitivity. Hence, there is a need to utilize other substitutes as a major ingredient in food commodities for individuals suffering from gluten sensitivity or celiac disease, without compromising consumer acceptability.

**Objective:**

The objective of the current study was to develop gluten-free cookies by partial substitution of the cookie’s rice flour with date fruit pulp and pit powder.

**Methods:**

Cookies were formulated by adding 100 g rice flour, 13.5 g eggs, 54 g sugar, 49.5 g shortening, and 2–3 drops of vanilla essence. Moreover, the proportion of date fruit pulp/date paste was 20, 40, 60, 80, and 100%, respectively, in groups T_1_, T_2_, T_3_, T_4,_ and T_5_. Similarly, the proportion of date pit powder varied from 5, 10, 15, 20, and 25%, respectively, in groups T_1_, T_2_, T_3_, T_4,_ and T_5_. Nutritional (proximate) composition, physical attributes, and sensory parameters of the prepared cookies were determined. Data were analyzed statistically using ANOVA and compared using Tukey’s Least Significance Difference (LSD).

**Results:**

The utilization of date paste and pit powder improved the proximate composition of cookies with increasing levels of added paste, whereas physical parameters showed a decrease in values with increasing levels of substitution. Organoleptic characteristics exhibited that biscuit quality was acceptable under various treatments.

**Conclusion:**

Rice-based cookies with added date fruit and pit powder could be a practical and acceptable substitute for individuals with gluten sensitivity.

## 1 Introduction

For a long time, bakery products have been consumed by humans as a staple diet, with cookies attaining the status of the most famous bakery item, offering delectable choices to consumers. Various bakery products are available in the market, but cookies occupy a major position due to their consumption by the masses and are a favorite food product, being inexpensive, affordable, shelf-stable, convenient, and savory (26). Owing to competition in the business sector and rising interest in natural, nutraceutical, and nutritious food products, efforts are being made to enhance the nutritional value of cookies. The shelf life and quality of cookies can be improved by the addition of various ingredients and variation in flour ratio (27). Cookies come under the category of confectionery items, which are dried to lower the moisture content and prepared from salt, flour, fat, preservatives, sweetening agents, and other ingredients (28, 29).

The bakery industry is constantly growing with novelty in techniques and food products. The development of healthier versions of cookies has also progressed significantly and holds a significant place for consumers with increased health concerns (1). Dried vegetables and fruit powder are among these sources with great health potential (30), and these have attained great upsurge in the recent era with main emphasis on functional food development with health-stimulating potential against various ailments like diabetes, cancer, ulcer, and atherosclerosis (2).

The gastrointestinal tract (GIT) is the primary organ system affected by celiac disease (CD), a chronic immune-mediated systemic disorder that also has extensive effects on other organ systems and poses serious health risks. Global population-based studies indicate that the overall seroprevalence of CD is approximately 1%, with a prevalence of 0.5% in Asian nations. Gluten-free (Rubin and Crowe, 2020). The clinical manifestations associated with celiac disease are very complex and remain undiagnosed in several cases. Rice flour is a healthy alternative for people on a gluten-free diet, with a rich source of B vitamins, proteins, potassium, calcium, niacin, fiber, iron, and thiamin (3). However, the actual prevalence of the disease seems to be much higher than what is documented in countries without screening programs. Unfortunately, due to a lack of comprehensive literature on CD that covers epidemiology, clinical presentation, and treatment, as well as limited resources, no research has been done to ascertain the true prevalence of CD in the Pakistani community (1).

celiac disease and non-celiac gluten sensitivity are often misunderstood as the same, but they are distinct clinical entities. Celiac disease is an autoimmune disorder triggered by gluten ingestion, leading to intestinal damage, and is diagnosed through serological tests and intestinal biopsy. In contrast, non-celiac gluten sensitivity presents with similar gastrointestinal symptoms but lacks autoimmune markers or intestinal damage (2).

Consumption of conventional wheat-based products is not recommended for individuals suffering from either gluten sensitivity or celiac disease. Therefore, there is a definite need for the replacement of wheat flour with some other suitable flour to ameliorate the risk associated with continuous consumption of wheat-based commodities. There has been an increase in demand for gluten-free products all over the globe (31). Gluten-free or low-gluten products are preferred by gluten-intolerant individuals (32). However, the use of the breeding process for the development of low-gluten wheat varieties is a difficult and time-consuming task (33, 34). Several flours could be employed for the substitution of wheat flour in gluten-free industries, but rice flour is one of the suitable alternatives used in the production of gluten-free or low-gluten diets (35). Rice flour has gained much popularity for the development of gluten-free commodities specifically for people with celiac disease (due to lower sodium, hypoallergenic, and mild flavor) (36). Production of rice-based cookies could be a suitable healthy alternative to wheat-based cookies (4).

Date palm is an edible sweet fruit of the Arecaceae family. Dates, being low in fat content, are beneficial for patients with heart-related diseases and can be consumed either through incorporation into food commodities or directly in their daily diet (37). Carbohydrate contents in dates are higher (approximately 70%) and are dominated mainly by sugars. The main sugar in dates is invert sugar, comprising fructose and glucose. Glucose is beneficial in providing instant energy (38), while fructose, being sweeter than glucose, creates a feeling of fullness among consumers, thereby lowering the overall caloric intake. Moreover, dates have a low-fat percentage (0.2–0.5%) and high protein content (2.3–5.6%). Furthermore, they are a very good source of several vitamins and minerals with significant amounts of antioxidants and phenolics. Utilization of date pit powder and date paste in cookies is a good alternative for simple sugars in cookies (39). The fruit of the date palm might be a suitable ingredient in cookies. Fructose and glucose, present in date palm, are utilized immediately after consumption as an instant energy source. There has been rising interest in today’s world in health-enhancing food products, promoting the fortification of nutritional constituents comprising antioxidant, fiber, protein, and active compounds in various food commodities with great potential (Vishwakarma et al., 2022). Therefore, this study aims to develop gluten-free cookies using rice flour as a safe alternative for individuals with gluten sensitivity, and to incorporate date palm as a natural sweetener to reduce reliance on refined sugar. This formulation not only addresses dietary restrictions but also promotes healthier snacking options with enhanced nutritional and functional properties.

### 1.1 Objectives

- Development of gluten-free cookies by substituting wheat flour with rice flour.
- Compositional analysis and physical characterization of rice-based cookies enriched with date paste and pit powder to study the effect of substitution of table sugar with natural sweetener (dates).
- Rice and date-based cookies assessment through sensory analysis.

## 2 Materials and methods

The current investigation was conducted at the National Agriculture Research Center (NARC), Islamabad. Accordingly, gluten-free cookies supplemented with date paste and pit powder were developed and characterized for their quality attributes and consumer acceptability. The methods and materials used are elaborated here.

### 2.1 Procurement of raw material

The rice flour and chickpeas were provided by the Food Science and Product Development Institute, NARC. Other ingredients (dates, sugar, baking powder, shortening, eggs, and vanilla essence) were purchased from the local market.

### 2.2 Cookies formulation

Cookies were prepared using the recipe provided in Table 1.

**Table 1:** Recipe for cookies formulation.

Ingredients	Quantity
Rice flour	100 g
Eggs	13.5 g
Sugar	54 g
Ghee/Shortening	49.5 g
Vanilla essence	2–3 drops

First, all the ingredients were weighed according to the recipe (5). Shortening was then manufactured by putting ghee in a microwave (1 min) and refrigerator (1 h) until its solidification. Ground sugar and shortening were mixed in a dough mixer (2 min) to obtain a creamy texture. Afterwards, date paste was put in a mixer along with the addition of sieved flour, date pits, baking powder, and mixed for 4–5 min. Dough was prepared by further addition of vanilla essence and egg in the prepared mixture by mixing (10–12 min) until formation of a viscoelastic dough ball. The spreading of the dough ball on the spreading sheet was done by roller, and uniformly shaped cookies were produced by dye. The cookies were put on the baking tray, greased with an oil brush, and the temperature of the oven was maintained at 180 °C for 10 min. The prepared cookies were put under controlled conditions for quality assessment and sensory analysis.

### 2.3 Treatment plan of cookies

Cookies were formulated by replacing rice flour with date pit powder at levels of 5, 10, 15, 20, and 25%, alongside the replacement of sugar at corresponding levels of 20, 40, 60, 80, and 100%, respectively. These formulations were compared with the control sample (T₀) to evaluate the impact of date pit powder substitution on the product’s quality.

500The relative proportion of all ingredients for the treatments is presented in Table 2.

**Table 2:** Treatment used in development of rice-based gluten-free cookies.

Ingredients	Rice flour (g)	Chickpea (g)	Date pits (g)	Date paste (g)	Sugar (g)
T_0_	80	20	0	0	100
T_1_	75	20	5	20	80
T_2_	70	20	10	40	60
T_3_	65	20	15	60	40
T_4_	60	20	20	80	20
T_5_	55	20	25	100	0

T_0_ = Control Sample_;_ T_1_= = Cookies with 75% rice, 5% date pits and 20% date paste; T_2_ = Cookies with 70% rice, 10% date pits and 40% date paste; T_3_ = Cookies with 65% rice, 15% date pits and 60% date paste; T_4_ = Cookies with 60% rice, 20% date pits and 80% date paste; T_5_ = Cookies with 55% rice, 25% date pits and 100% date paste it’s with 80% rice, without date pits and date powder.

### 2.4 Proximate analysis of cookies

#### 2.4.1 Moisture determination

The cookies were assessed for moisture content using the AOAC method no. 934–10 (Equation 1). Accordingly, a 10 g biscuit sample was dried at 105° C in the hot-air oven (Model: DO-1-30-02, PCSIR, Pakistan) until a constant weight was obtained.


(1) $$\mathrm{Moisture}=\frac{\begin{array}{l} \;\mathrm{weight of sample before drying}-\\ \mathrm{weight of sample after drying} \end{array}}{\mathrm{weight of sample before drying}}\times 100$$

#### 2.4.2 Determination of ash

The ash content in the gluten-free biscuit was measured by sample incineration using the procedure described by AOAC (2006) Method No. 942–05 (Equation 2). Purposely, a 5 g sample was put in a crucible and charring was done over a flame until the complete disappearance of the fumes. Then, ignition of the samples was performed in a muffle furnace (MF-1-02, PCSIR, Pakistan) at a temperature of 550–600 °C. for 5–6 h until formation of grayish white residues. The percentage ash was calculated by the mathematical equation given below:


(2) $$\mathrm{Ash}=\frac{\mathrm{weight of}\;\mathrm{ash}}{\;\mathrm{Weight of sample}}\times 100$$

#### 2.4.3 Crude fiber analysis

The crude fiber content of the gluten-free cookies was ascertained by Method no. 978–10 following the guidelines of AOAC (2006) (Equation 3). Accordingly, about 2 g sample of cookies was digested in the Fiber Tech apparatus (Labconco Corporation, Kansas, United States). for 30 min by 1.25% sulfuric acid (200 mL). The H_2_SO_4_ was drained and washed three times following filtration of the digested sample. The washing was done thrice using boiled distilled water to obtain the alkali-free sample. The acid-free residue was then boiled in 1.25% sodium hydroxide (NaOH) solution for 30 min to solubilize protein and lignin. This was followed by filtration and three washes using boiled distilled water to obtain the alkali-free sample. The obtained residues were weighed (W_1_) and dried at 130 °C for 2 h. The ignition of the obtained sample was performed in a muffle furnace (MF-1/02, PCSIR, Pakistan) at 550–650 °C until the formation of white ash, and was weighed again (W_2_). The crude fiber percentage in the date sample was determined by following the expression:


(3) $$\mathrm{Crude Fibre}=\frac{W1-W2}{\mathrm{Sample weight}}\times 100$$

#### 2.4.4 Crude fat analysis

The crude fat was assessed by AOAC (2006) method No. 920–39 (Equation 4). Purposely, a 2 g sample was weighed in a thimble. Afterwards, n-hexane (50 mL) was added to a flask attached to a Soxhlet (Model: H-21045 Extraction Unit, Hoganas, Sweden). The extraction of the fat content of the sample was done (2–3 h) by regulation of flow rate (3–4 drops) /second of hexane. Following 6–7 siphon back, the thimble was removed and subjected to drying in a hot-air oven (105 °C) for 1 h and again weighed by an electric balance.


(4) $$\mathrm{Crude}\;\mathrm{Fat}=\frac{\mathrm{Weight of hexane extract}}{\mathrm{Weight of sample}}\times 100$$

#### 2.4.5 Crude protein analysis

Percent nitrogen was determined by the method No. 984–13 of AOAC (2006) (Equation 5). In this context, a 500 mg biscuit sample was digested by concentrated H_2_SO_4_ using a digestion mixture (K_2_SO_4_: FeSO_4_: CuSO_4_:100:5:10) until a light green color (3–4 h) appeared. Following digestion, the sample was put into a volumetric flask (250 mL) and diluted to volume with distilled water. For distillation, 10 mL of the digested sample and 10 mL of 40% sodium hydroxide (NaOH) were added to the distillation apparatus. The liberated ammonia was collected in a beaker containing 4% boric acid solution with methyl red indicator. The boric acid acted as a receiver solution, reacting with the ammonia to form ammonium borate, which caused a color change indicating ammonia capture. The amount of ammonia, and thus nitrogen, was determined by titrating the distillate against 0.1 N H₂SO₄ until a light golden color endpoint was reached. Crude protein content was then calculated by multiplying the percentage of nitrogen by a factor of 5.80.


(5) $$\mathrm{Nitrogen}\;\left(\%\right)=\frac{\begin{array}{l} \mathrm{Volume of}\;0.1\;N\;\mathrm{sulphuric}\\ \mathrm{acid used}\times 0.0014\times 250 \end{array}}{\;\mathrm{Sample weight}\times \mathrm{Aliquot volume}}\times 100$$

Crude Protein=Nitrogen%×5.80

#### 2.4.6 Determination of nitrogen free extract

For analysis of NFE, the AOAC (2000) protocol was used (Equation 6), and calculation of carbohydrates was done by the difference between 100 and the sum of other proximate parameters as Nitrogen-free extract (NFE) percentage of carbohydrate.


(6) $$\mathrm{Carbohydrate}\%=100-\left(\begin{array}{l} \mathrm{Moisture}+\mathrm{Protein}+\mathrm{Fat}+\\ \mathrm{Ash}+\mathrm{crude fiber} \end{array}\right)$$

#### 2.4.7 Determination of sugar

The sugar content in the prepared date fruit and pit powder-enriched biscuit was determined by the methods described by (6). The date sample was weighed to be exactly 5 g and refluxed with 25 mL HPLC-grade water. The homogenization of the mixture was done for 5 min at 280 rpm by orbital shaker and allowed to rest for 2 h. Then it was centrifuged at 5000 rpm for 10 min at 4 °C, followed by filtration, using 0.45 μm membrane filters. Then, quantification of the reducing and non-reducing sugars in the date sample was done by HPLC. Afterwards, separation was done at room temperature by Lichorospher® 100 NH2 5 μm column. Texture analysis of the cookies was performed using a Texture Analyser (Model TVT-300XP) available at PCSIR Laboratories, Pakistan. Ultrapure water and Acetonitrile (80/20 v/v) constitute the mobile phase. A connection between the liquid chromatographic system and the refractive index detector 10 A was made. The flow rate was adjusted to 0.8 mL/min, and an injection volume of 20 mL was set in the analysis. The calibration of the integrator was done by external standards comprising fructose 2%, glucose 2 and 1% sucrose. The calculation of the total reducing sugar was done by the addition of the fructose and glucose contents.

### 2.5 Physical analysis

The vernier caliper was used to measure the width/diameter of the cookies (calculated twice by rotation of the product at 90 °C). The measurement of the thickness was done by stacking cookies on one another and dividing the total e by six to obtain the mean value. The calculation of the spread ratio was done by dividing the thickness value shown as diameter/thickness (Akin et al., 2024). The texture analyzer (Model) was used to assess the hardness of date cookies by utilizing the three-point bend ring methodology (1.0 mm/s pretest speed, 5 kg load cell, test speed 1.0 mm/s, and posttest speed 10.0 mm/s with a distance of 10 mm).

### 2.6 Sensory evaluation of cookies

The sensory evaluation of the cookie sample was performed by the methods of (7, 8). The presentation of the sample was done in a homogeneous pattern by using identical conditions, provided with a soft lighting environment. The evaluation of appearance, color, texture, aroma, and taste was done. The average value was taken and used as an indication of overall acceptability. For neutralizing taste between different treatments, salted crackers along with mineral water were used. A trained panel of 10 members, comprising staff from NARC and PCSIR, conducted the organoleptic evaluation. Average scores were used to determine overall acceptability. The assessment was made based on a nine-point hedonic scale, 1 representing “disliked extremely” and 9 “liked extremely.” The comparison of the date pit and paste fortified sample was done with a control for assessing overall acceptance and quality by consumers.

### 2.7 Statistical analysis

Data acquired from each analysis were subjected to statistical analysis to check the level of significance. Statistical model (Statistix 8.1) was used, and data were analyzed by using a completely randomized design (CRD). A further comparison of mean values was done by Tukey’s LSD test.

## 3 Results and discussion

### 3.1 Nutritional composition

Proximate composition of cookies made from rice cookies supplemented with date pit and fruit powder exhibited that moisture content ranged between 4.45–10.25% (Table 3). The rise in moisture content in various samples was possibly due to the rising concentration of date paste, since it contained more moisture. These values were higher than the control sample (without date pit and fruit powder), and from the results of (9) who reported moisture between 2.0 to 3.8% in cookies supplemented with 5 and 10% date fruit powder.

**Table 3:** Proximate analysis of rice enriched cookies fortified with date paste and pit powder.

Treatments	Fiber%	Ash%	Moisture%	Fat %	Protein %	NFE %
T_0_	1.6 ± 0.07^f^	1.28 ± 0.06^d^	3.32 ± 0.15^f^	77.00 ± 3.16 ^a^	10.14 ± 0.42^b^	6.66±
T_1_	2.44 ± 0.1^e^	1.42 ± 0.06^cd^	4.45 ± 0.2^e^	77.70 ± 3.5^a^	10.62 ± 0.48^b^	3.37±
T_2_	3.58 ± 0.16^d^	1.64 ± 0.07^bc^	7.85 ± 0.36^c^	78.00 ± 3.51 ^a^	11.21 ± 0.52^b^	2.28±
T_3_	4.70 ± 0.22^c^	1.76 ± 0.08^b^	6.73 ± 0.30^d^	79.10 ± 3.64^a^	11.63 ± 0.56^b^	3.92±
T_4_	6.55 ± 0.09^b^	1.84 ± 0.09^b^	9.17 ± 0.38^b^	80.50 ± 3.54^a^	13.91 ± 0.67^a^	11.97±
T_5_	9.80 ± 0.46^a^	2.45 ± 0.11^a^	10.25 ± 0.46^a^	81.00 ± 3.89^a^	14.44 ± 0.59^a^	17.74±
*F*-value	529**	78.0**	204**	0.61^NS^	31.7**	

T_0_ = Control Sample_;_ T_1_= = Cookies with 75% rice, 5% date pits and 20% date paste; T_2_ = Cookies with 70% rice, 10% date pits and 40% date paste; T_3_ = Cookies with 65% rice, 15% date pits and 60% date paste; T_4_ = Cookies with 60% rice, 20% date pits and 80% date paste; T_5_ = Cookies with 55% rice, 25% date pits and 100% date paste it’s with 80% rice, without date pits and date powder. Values show mean ± SD; one-way ANOVA followed by Turkey’s HSD multiple comparison Tests. *Significant, **Highly significant; NS, Non-significant.

The fiber content of several types of cookies was 2.44–9.80%, which was higher than that of the control, which was 1.6%. These values were higher/greater than the 3.16–3.95% reported by (10). However, researchers reported a similar rise in fiber content with a corresponding increase in date powder due to the high fiber content of the date powder. Mean values for fat content of cookies enriched with various quantities of date pit powder ranged from 77 to 81% (Table 3), with a maximum score in T_5_ (81.00%) and a minimum for the control sample (77%). Crude fat in date pit and paste-enriched cookies ranged between 77 and 81% with maximum quantity in T_0_ (Control Sample) and minimum in T_5_ (Cookies with 55% rice, 25% date pits, and 100% date paste it is with 80% rice, without date pits and date powder). Cookies showed higher fat content due to enrichment with date paste and date pit powder, with a gradual increase observed as the concentration of these ingredients increased. While this enhancement contributes to the nutritional richness of the product, the relatively high fat content raises potential health concerns. Therefore, future studies should investigate the quality and type of fat present in date-incorporated foods to determine their impact on overall health and suitability in functional food development. The results revealed an increase in fat content with an increase in concentration of date pit powder and date fruit. Results showed significant variation in fat content with the addition of date pit powder and date paste. These results are in line with the findings of (11) who reported a significant rise in fat content of bars with the addition of date pit powder and soy protein isolate.

As shown in the results, maximum ash content was 2.45% in T_5_ in cookies prepared from 100% date paste and 25% date pit powder, trailed by 1.84% ash in T_4_ (Cookies with 60% rice, 20% date pits and 80% date paste) with 80% date paste and 20% date pit powder. The results demonstrated significant variation of ash content in various treatments (*p* < 0.005). Results are in close conformity with (12, 13), who reported a rise in ash level of biscuits with rising concentration of date powder, with a maximum value in 100% date powder-enriched biscuits. Protein content of biscuits was reported between (10.14–14.44%), which is similar to the findings of (14).

The sugar level increased with increasing levels of date pit and paste, with a minimum amount in control (T_0_) and a maximum in T_5_ with 100% date paste. The reason was obviously the rising concentration of date powder, which contributed to higher sweetness in the cookies. These results are in line with (15) since they also reported a similar rising trend of carbohydrates with increasing concentration of date paste-enriched cookies. However, the results are antagonistic to the findings of (16) whereby they reported a decrease in carbohydrate content with rising date pit powder concentration. This contrary behavior might be the result of a parallel increase in date paste that overcame the bitterness that might be contributed by date pit powder in various treatments of cookies.

The findings of the present study are consistent with previous research aimed at developing gluten-free bakery products using alternative flours and natural sweeteners. Similar to the results reported by (17). Moreover, the use of date pit powder as a partial sugar and flour substitute aligns with the work of (18), who highlighted the nutritional benefits of date by-products, including high fiber, antioxidants, and natural sugars. Our study extends this approach by showing that date pit powder can improve the nutritional value of gluten-free cookies without compromising acceptability. In line with (19), who formulated gluten-free cookies with fruit-based sweeteners, our results further support the potential of using naturally sweet, fiber-rich ingredients in developing functional snacks. Overall, this research contributes to the growing body of evidence supporting the use of locally available, health-promoting ingredients in the formulation of gluten-free products that meet both dietary needs and consumer expectations.

Particularly in gluten-free and high-fiber dietary applications, the nutritional makeup of the date pastes and date pit-enriched cookies underscores their potential as useful and health-promoting snacks. Date by-products are a nutrient-dense ingredient that contributes to the notable increase in dietary fiber, protein, and ash content. Increased protein content promotes muscle maintenance and satiety, two crucial aspects of snack design, while higher fiber levels may help with digestive health. Future research should examine the lipid profile to determine the health implications of this enhancement, even though the fat content also increased. This improvement adds to energy density and texture. In order to create reasonably priced and nutritionally enhanced bakery goods, it is beneficial to use locally accessible, underutilized ingredients like fruit paste and date pits, as evidenced by the observed nutritional improvements. These findings are particularly relevant for regions where nutrient deficiencies are common and cost-effective functional foods are needed.

### 3.2 Physical analysis of cookies

Results of physical attributes of control and date-enriched rice cookies are compared in Table 4. Thickness, diameter, width, and spread ratio were significantly affected by the addition of date pit powder and paste. A rise in diameter from 51.99 to 39.66 was observed; however, a decline was observed in values of the spread ratio and spread factor with increased level of date powder. Spread ratio is an important factor in assessing cookie quality, and higher values are more desirable. These results are similar to (6) for cookies prepared by the addition of date fruit powder. Spread ratio and percent declined with rising concentration of date pit powder and paste. The competition of ingredients for available moisture/water was the main contributing factor for variation of the spread ratio; it could have also been influenced by various functional properties of fat and protein contents (20) The competition of ingredients for available moisture might not be the contributing factor for the variation of the spread ratio in the current case, since date paste and pit powder, as well as rice absorbed water in the dough mixing process (9). Hence, it can be concluded that fat and protein contents influenced the spread ratio of the cookies in the current study (Table 5).

**Table 4:** Sugar contents in rice enriched cookies supplemented with date paste and pit powder.

	Sugar %		
Treatments	Reducing	Non-reducing	Total Sugar
T_0_	0.0^d^	54.00 ± 2.21^a^	54.00 ± 2.59^a^
T_1_	12.43 ± 0.46^c^	19.75 ± 0.91^c^	32.18 ± 1.48^c^
T_2_	12.41 ± 0.57^c^	14.93 ± 0.67^d^	27.36 ± 1.26^c^
T_3_	13.68 ± 0.66^c^	27.36 ± 1.12^b^	41.04 ± 1.68^b^
T_4_	25.33 ± 1.09^b^	20.27 ± 0.87^c^	45.60 ± 2.05^b^
T_5_	47.12 ± 2.26^a^	6.840 ± 0.28^e^	53.96 ± 2.59^a^
*F*-value	638**	576**	90.8**

T_0 = Control Sample;_ T_1_= = Cookies with 75% rice, 5% date pits and 20% date paste; T_2_ = Cookies with 70% rice, 10% date pits and 40% date paste; T_3_ = Cookies with 65% rice, 15% date pits and 60% date paste; T_4_ = Cookies with 60% rice, 20% date pits and 80% date paste; T5 = Cookies with 55% rice, 25% date pits and 100% date paste it’s with 80% rice, without date pits and date powder. Values show mean ± SD; one-way ANOVA followed by Turkey’s HSD multiple comparison Tests. *Significant; **Highly significant; NS, Non-significant.

**Table 5:** Physical analysis of cookies.

Treatment	Thickness/height (mm)	Width/Diameter (mm)	Spread ratio	Spread factor
T_0_	6.44 ± 0.06^b^	51.99 ± 2.34^a^	8.07 ± 0.36^a^	100 ± 4.5^a^
T_1_	6.45 ± 0.26^b^	46.55 ± 2.05^b^	7.22 ± 0.29^b^	89 ± 3.65^b^
T_2_	6.85 ± 0.31^b^	43.32 ± 1.78^bc^	6.32 ± 0.29^c^	78 ± 3.2^c^
T_3_	7.22 ± 0.35^b^	40.10 ± 1.88^c^	5.55 ± 0.24^d^	68 ± 2.99^d^
T_4_	8.33 ± 0.37^a^	39.99 ± 1.76^c^	4.80 ± 0.22^de^	59 ± 2.71^de^
T_5_	8.55 ± 0.37^a^	39.66 ± 1.82^c^	4.64 ± 0.21^e^	57 ± 2.66^e^
*F*-value	27.4**	19.0**	73.1**	78.8**

T_0_ = Control Sample_;_ T_1_= = Cookies with 75% rice, 5% date pits and 20% date paste; T_2_ = Cookies with 70% rice, 10% date pits and 40% date paste; T_3_ = Cookies with 65% rice, 15% date pits and 60% date paste; T_4_ = Cookies with 60% rice, 20% date pits and 80% date paste; T_5_ = Cookies with 55% rice, 25% date pits and 100% date paste it’s with 80% rice, without date pits and date powder. Values show mean ±SD; one-way ANOVA followed by Turkey’s HSD multiple comparison Tests. *Significant; **Highly significant; NS, non-significant.

The spread ratio is the assessment of flour’s ability to rise and is an ultimate measure of the flour quality (21). Cookies with a great spread ratio are more desirable. Texture is an important and desirable parameter of cookies, whereas hardness is the peak force necessary for biscuit breakage. A similar rise in thickness and width has been reported by (22) in cookies produced by a blend of pigeon pea, cooked banana, and sweet potato.

### 3.3 Sensory evaluation

Color is vital for arousing an individual’s appetite and is used for process control in roasting and baking since brown pigments are produced during the reaction process (23). In our study, the color of cookies changed from brown to dark brown with increasing levels of inclusion of date paste and pit powder. The reason for this color variation might have been the composition of ingredients, oven air velocity, and red pigmentation produced from nonenzymatic browning reactions or the Maillard reaction. The present results are similar to the findings of other researchers (24).

Results for sensory attributes are presented in Table 6. There was a significant difference in color, flavor, taste, texture, and overall acceptance. The taste and texture were influenced greatly by various levels of date pit powder and date paste. Cookies with greater levels of added date pit powder and date paste exhibited higher scores in terms of color, taste, flavor, and overall acceptability. Taste has a vital role in the acceptance of a specific product, and its score increases with the increasing level of added date powder. Similar results for sensory parameters were reported by (25) in the production of cookies enriched with unripe cooking banana, sweet potato, and pigeon pea (Table 7).

**Table 6:** Sensory quality of all treatments of cookies.

Treatment	Color	Taste	Flavor	Texture	Overall acceptance
T_0_	7	6	7	5	6
T_1_	7	7	6	6	7
T_2_	6	6	6	5	6
T_3_	6	8	7	6	6
T_4_	7	6	8	6	7
T_5_	6	7	6	5	7

T_0_ = Control Sample_;_ T_1_= = Cookies with 75% rice, 5% date pits and 20% date paste; T_2_ = Cookies with 70% rice, 10% date pits and 40% date paste; T_3_ = Cookies with 65% rice, 15% date pits and 60% date paste; T_4_ = Cookies with 60% rice, 20% date pits and 80% date paste; T_5_ = Cookies with 55% rice, 25% date pits and 100% date paste it’s with 80% rice, without date pits and date powder.

**Table 7:** Mean squares for sensorial parameters of rice-based cookies enriched with date.

SOV	df	color	Taste	Flavor	Texture	Overall acceptability
Treatments	5	1.25^NS^	1.55^NS^	1.12^NS^	1.15^NS^	0.99^NS^
Error	12	0.44	0.78	1.00	0.72	0.78

NS, Non-significant.

## 4 Conclusion

The addition of date fruit paste and date pit powder to rice flour-based cookies significantly improved their nutritional profile, especially in terms of fiber, protein, and ash content. These improvements point to the potential of date-derived ingredients in functional, gluten-free bakery products. Although higher levels of date enrichment reduced certain physical properties such as spread ratio and diameter, the sensory evaluation revealed that all treatments were highly acceptable to consumers. This balance of nutritional enhancement and organoleptic quality promotes the development of functional snacks for people who are gluten sensitive or have celiac disease. Overall, the study emphasizes the potential of locally available ingredients such as rice flour and date by-products in developing affordable, health-conscious food options that are in line with current dietary trends and public health needs.

## Data Availability

The raw data supporting the conclusions of this article will be made available by the authors, without undue reservation.
